# Coordination-assembled myricetin nanoarchitectonics for sustainably scavenging free radicals

**DOI:** 10.3762/bjnano.13.23

**Published:** 2022-03-01

**Authors:** Xiaoyan Ma, Haoning Gong, Kenji Ogino, Xuehai Yan, Ruirui Xing

**Affiliations:** 1 Graduate School of Bio-Applications and Systems Engineering, Tokyo University of Agriculture and Technology, Tokyo, Japan; 2 State Key Laboratory of Biochemical Engineering, Chinese Academy of Sciences, Institute of Process Engineering, Beijing, P. R. China; 3 School of Chemical Engineering, University of Chinese Academy of Sciences, Beijing, P. R. China

**Keywords:** antioxidant, co-assembly, glutathione, myricetin, nanoarchitectonics

## Abstract

Oxidative stress can lead to permanent and irreversible damage to cellular components and even cause cancer and other diseases. Therefore, the development of antioxidative reagents is an important strategy to alleviate chronic diseases and maintain the redox balance in cells. Small-molecule bioactive compounds have exhibited huge therapeutic potential as antioxidants and anti-inﬂammatory agents. Myricetin (Myr), a well-known natural flavonoid, has drawn wide attention because of its high antioxidant, anti-inflammatory, antimicrobial, and anticancer efficacy. Especially regarding antioxidation, Myr is capable of not only chelating intracellular transition metal ions for removing reactive oxygen species, but also of activating antioxidant enzymes and related signal pathways and, thus, of sustainably scavenging radicals. However, Myr is poorly soluble in water, which limits its bioavailability for biomedical applications, and even its clinical therapeutic potential. The antioxidant peptide glutathione (GSH) plays a role as antioxidant in cells and possesses good hydrophilicity and biocompatibility. However, it is easily metabolized by enzymes. To take advantages of their antioxidation activity and to overcome the abovementioned limitations, GSH, Zn^2+^, and Myr were selected to co-assemble into Myr-Zn^2+^-GSH nanoparticles or nanoarchitectonics. This study oﬀers a new design to harness stable, sustainable antioxidant nanoparticles with high loading capacity, high bioavailability, and good biocompatibility as antioxidants.

## Introduction

Oxidative stress, caused by an imbalance between antioxidative and oxidative systems, leads to permanent and irreversible damage of cellular components, such as proteins, lipids, and nucleic acids [1]. Furthermore, oxidative stress leads to diseases including Alzheimer’s disease [2], cardiac disease [3], atherosclerosis [4], kidney disease [5], sepsis [6], cancer [7], and inflammatory diseases (e.g., periodontal disease and inflammatory bowel disease) [8–9]. Therefore, the development of antioxidative reagents is a crucial strategy to alleviate chronic diseases and maintain the redox balance.

Increasingly efficient antioxidant materials are used for effectively scavenging multiple ROS. Metal-based nanomaterials, such as CeO_2_ and Fe_3_O_4_, have been widely applied for antioxidant therapy [10]. In addition, bioactive small-molecule compounds, such as bilirubin and curcumin, and antioxidant peptides such as glutathione (GSH) and casein phosphopeptides, exhibited huge therapeutic potential in antioxidant treatments [11–13]. Nevertheless, a plenty of disadvantages restrict biomedical applications, namely low biocompatibility of the metal-based nanomaterials, low bioavailability of hydrophobic small-molecule compounds, and easy degradation of antioxidant peptides by proteases. The combination of liposomes or polymers with different payload materials has been reported, for example, PEG-modified liposomes loaded with resveratrol, layer-by-layer-coated gelatin nanoparticles, or Gelucire-based solid lipid and polymeric micelles [14–19]. However, low loading efficiency, systemic toxicity, and tedious preparation processes hinder biomedical applications.

Myricetin (Myr), a well-known natural flavonoid, has drawn wide attention because of its high antioxidant, anti-inflammatory, antimicrobial, and anticancer efficacy [16]. Myr is capable of not only chelating intracellular transition metal ions for removing reactive oxygen species (ROS) [20], but also of activating antioxidant enzymes and the AMPK/NRF2 signal pathway [21], yielding sustainable scavenging of radicals. Myr can inherently increase body resistance to carcinogens, viruses, and allergens [17]. In spite of the tremendous potential, Myr possesses the same shortcomings as many hydrophobic small molecules, namely low bioavailability, poor water solubility and rapid degradation at pH > 6.8, which limits its clinical therapeutic potential [22]. GSH consists of glycine, cysteine, and glutamic acid. The cysteine residue plays a pivotal role in protecting the body from oxidation damage; however, GSH is easily metabolized by enzymes [23]. In this work, we employed a facile co-assembly strategy to design hybrid nanoparticles as antioxidants [24–31]. Myr, Zn^2+^, and GSH were co-assembled to Myr-Zn^2+^-GSH (MZG) nanoparticles. The obtained MZG nanoparticles exhibit high loading capacity as well as good bioavailability and biocompatibility, leading to stable antioxidant effects.

**Figure 1:**
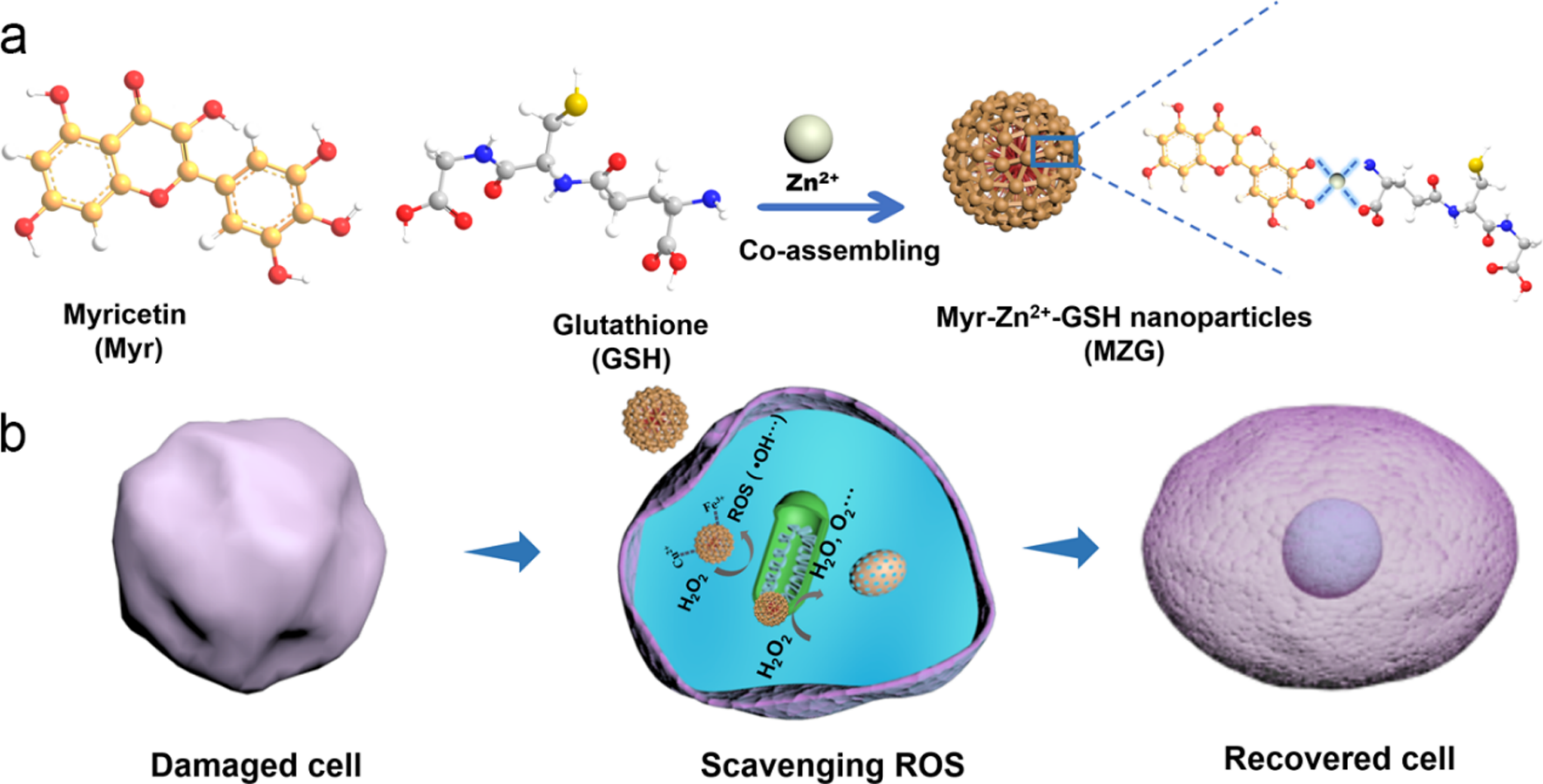
Schematic illustration of preparing MZG nanoparticles (a) and (b) antioxidation mechanism in cells. MZG nanoparticles afford the antioxidant activity for maintaining redox homeostasis in cells.

## Experimental

### Synthesis of MZG

10 mg·mL^−1^ of Myr was dispersed in 0.1 M NaOH, 10 mg·mL^−1^ of GSH was dispersed in deionized water, and a 100 mM solution of Zn^2+^ was prepared. MZG nanoparticles were produced by adding 100 µL of Myr into a mixed solution of 200 µL of GSH solution, 15.7 µL of Zn^2+^ solution and 684 µL of water. The Myr solution should be freshly prepared for use due to precipitation. The freshly prepared MZG nanoparticles were aged for 24 h at room temperature and placed in the dark. As-prepared nanoparticles were concentrated, purified by centrifugation, and used for further experiments.

### Quantitative analysis of MZG

The precipitates of MZG were collected after centrifuging and washing with water and, subsequently, dissolved in 0.1 M NaOH solution. The concentrations of Myr and GSH were determined by UV–vis spectroscopy. The concentration of Zn^2+^ was measured by inductively coupled plasma optical emission spectroscopy (ICP-OES).

### Evaluation of ROS scavenging activity

First, a solution of 2,2′-azino-bis(3-ethylbenzothiazoline-6-sulfonic acid) radical cations (ABTS^+^) solution was prepared. 7 mM of ABTS solution was mixed with 2.45 mM of potassium persulfate solution and kept for 12 h in the dark. The UV–vis absorption intensity of a 0.7 mM ABTS solution (diluted in PBS) was 0.7 ± 0.02. A certain volume of diluted ABTS^+^ solution was added to different concentrations of Myr, GSH, and MZG. A UV–vis spectrophotometer was used to record the absorption intensity of ABTS^+^ at 734 nm. The radical scavenging rate was calculated according to the following equation:




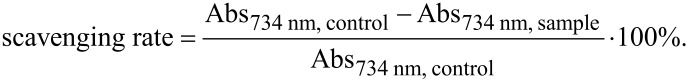




MZG nanoparticles and Myr/GSH complex were dispersed in H_2_O for 5 days. Diluted ABTS solution was added with the same concentration of MZG and Myr/GSH complex (equivalent concentration of Myr and GSH: 4 μg·mL^−1^ and 8 μg·mL^−1^, respectively) every day. The UV–vis absorption intensity of ABTS at 734 nm was measured, and the radical scavenging rate was calculated.

1 mL of MZG suspension (equivalent concentration of Myr and GSH: 4 μg·mL^−1^ and 8 μg·mL^−1^, respectively) and 1 mL of water as control were added to the same volume of ABTS^+^ solution. UV–vis spectra and the absorption intensity at 734 nm were recorded. The absorption intensity of ABTS^+^ at 734 nm was measured again after 24 h. Then, the same volume of ABTS^+^ solution was added to the abovementioned MZG suspension and H_2_O, and UV–vis spectra and the absorption intensity at 734 nm were recorded. Before adding the same volume of ABTS^+^ solution, UV–vis spectra and the absorption intensity at 734 nm were recorded. This was repeated for 5 days.

The as-prepared MZG nanoparticles were acquired by centrifugation. Then MZG nanoparticles were incubated with diﬀerent concentrations of H_2_O_2_ (0.01, 0.1, 1, 10, and 100 mM). UV–vis spectrophotometry and dynamic light scattering were used to record absorption spectra and size change.

### Cytotoxicity experiment in vitro

The cytotoxicity of the as-prepared MZG nanoparticles was assessed against 3T3 cells. 3T3 cells were cultivated with Dulbecco's Modified Eagle Medium (DMEM) containing 10% (v/v) fetal bovine serum (FBS) and 1% (v/v) penicillin/streptomycin. 3T3 cells at a density of 1 × 10^5^ cells per well were incubated with different concentrations of MZG (equivalent concentration of Myr: 0, 10, 20, 40, 80, and 100 µM) for 24 h. The cell viability was tested with the methyl thiazolyl tetrazolium (MTT) assay.

### Evaluation of ROS scavenging in cells

First, the median lethal dose (LD_50_) value of H_2_O_2_ was evaluated. 3T3 cells at a density of 2 × 10^5^ cells per well were incubated with H_2_O_2_ at different concentrations (0, 10, 20, 40, 60, 80, 100, 150, and 200 µM). Second, the ROS scavenging activity of MZG was evaluated. After the treatment of 3T3 cells with different concentrations of MZG for 24 h, 100 µM of H_2_O_2_ was used to treat the 3T3 cells. The capability of protecting cells from damage was accessed by the cell viability assay. After that, 2′7′-dichlorodihydrofluorescein diacetate (DCFH-DA) dye was used to incubate these cells for 5 min and the fluorescence intensity of the cells was recorded via confocal laser scanning microscopy.

## Results and Discussion

### Synthesis and characterization of MZG

**Figure 2:**
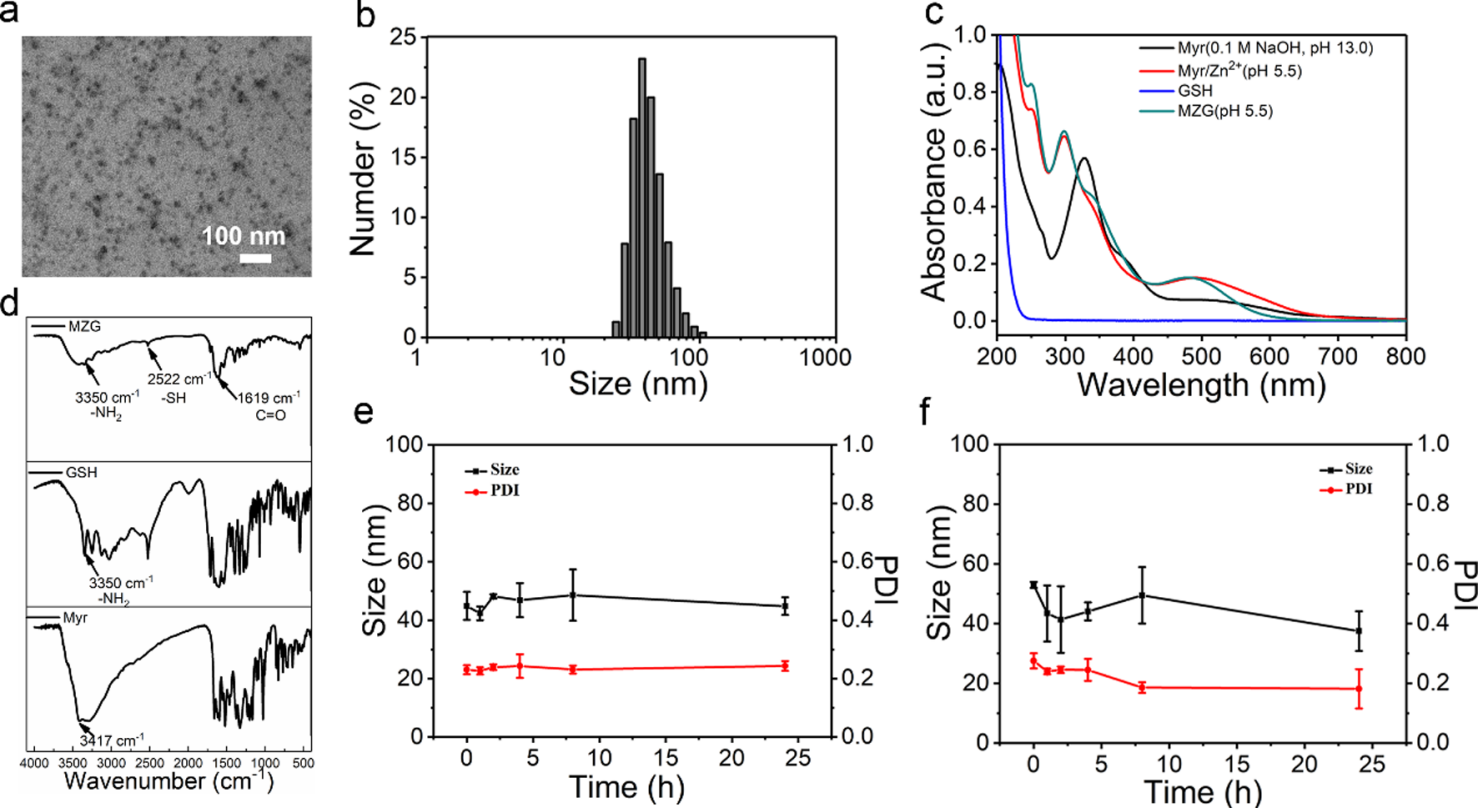
Physicochemical characterization of MZG nanoparticles. (a) TEM image. (b) DLS proﬁle of MZG nanoparticles. (c) UV–vis absorbance spectra of Myr dissolved in (0.1 M NaOH), Myr/Zn^2+^ complex, GSH, and MZG (equivalent concentration of Myr: 20 μg·mL^−1^). (d) FTIR spectra of MZG, GSH, and Myr. (e) Stability evaluation of MZG nanoparticles during incubation in aqueous solution at 37 °C for 24 h. (f) Stability evaluation of MZG nanoparticles during incubation in the mixture medium of PBS containing 9% DMEM and 1% FBS (v/v) at 37 °C for 24 h (equivalent concentration of Myr: 0.5 mg·mL^−1^).

We chose Zn^2+^, a typical essential metal, to effectively bond Myr and GSH via coordination interaction (Myr/GSH = 1:2) [32–34]. The nanoparticles were formed by coordination self-assembly of Zn^2+^, Myr, and GSH (Figure 1a). They were expected to show good antioxidant activity to protect cells from the ROS-induced damage (Figure 1b). The transmission electron microscopy (TEM) image in Figure 2a shows spherical MZG nanoparticles. Size and zeta potential value of the MZG nanoparticles were 44.6 ± 26.5 nm and −23.1 ± 3.4 mV, respectively, measured by dynamic light scattering (DLS) (Figure 2b) and consistent with the TEM results. Importantly, the co-assembly mechanism was further revealed through quantitative stoichiometry analysis. The quantitative component analysis by UV–vis and ICP-OES proved that the molar ratio of Myr/GSH/Zn^2+^ was close to 1:1:1 (Supporting Information File 1, Table S1 and Figure S1).

The UV–vis absorption spectra of pure Myr dispersed in 0.1 M NaOH (pH 13), Myr/Zn^2+^ complex (pH 5.5), and MZG nanoparticles (pH 5.5) were measured. The UV–vis absorption spectrum of MZG nanoparticles exhibited a blue shift at 550 nm, compared with Myr/Zn^2+^, which was assigned to the charge transfer between GSH and Zn^2+^ (Figure 2c). These results demonstrated that Zn^2+^ as the coordination component co-assembles with Myr and GSH. Fourier-transform infrared (FTIR) spectra were used to further confirm the self-assembly of the MZG nanoparticles. In Figure 2d, the two bands at 2522 cm^−1^ and 3350 cm^−1^ were assigned to the mercapto group (–SH) and the stretching vibration of the amino group (–NH_2_) of GSH. The band at 1619 cm^−1^ was assigned to the C=C group of Myr. In addition, the band at 3417 cm^−1^ was assigned to the phenolic hydroxy group. The two bands of phenolic hydroxy and amino groups shifted to lower wavenumbers, compared with the corresponding bands of Myr and GSH, suggesting that these groups coordinated to Zn^2+^.

The co-assembly approach mitigates the poor water solubility of Myr and improves its bioavailability for further biomedical applications. The stability of the co-assembled nanoparticles is important for antioxidant application. The DLS proﬁles were used to evaluate the stability of the MZG nanoparticles. The MZG nanoparticles (0.5 mg·mL^−1^) were either dispersed in water or diluted 10-fold (v/v) in DMEM containing 10% (v/v) FBS at 37 °C for 24 h to investigate the stability. The change of DLS was recorded at different time points (0, 2, 4, 8, 24, 48, and 72 h), showing that the average size and size distribution did not change over time (Figure 1e,f). The results indicate that MZG nanoparticles are stable in water and culture medium. Although noncovalent interactions are relatively weak compared to covalent interactions, the metal coordination interaction is the strongest noncovalent interaction [4]. Hence, this interaction assured the stability of assembled nanoparticles in physiological conditions.

### Evaluation of ROS scavenging activity

Myr possesses excellent antioxidant activity to scavenge ROS, which has impact on chelate metal ions such as Fe^2+^ and Cu^2+^, inhibits glutathione reductase activity, and regulates PI3K/Akt and MAPK signal pathways to avoid oxidative stress-induced apoptosis [20,35–37]. Also, GSH acts as an important antioxidant in the body owing to the –SH group, which can be reduced. GSH is a non-enzymatic antioxidant molecule, which is necessary for cell redox homeostasis and survival [38].

**Figure 3:**
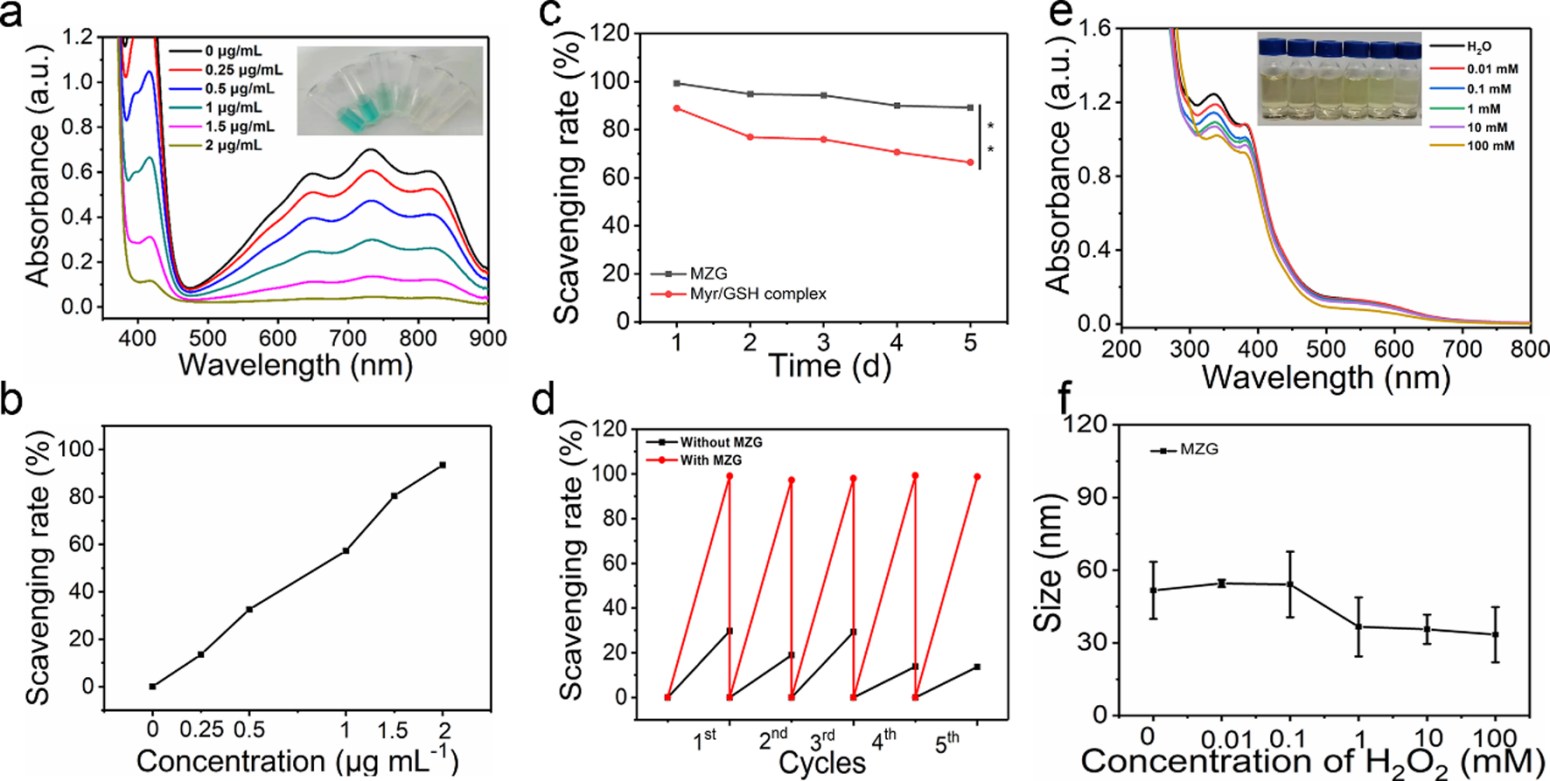
Measurement of ROS scavenging. (a) UV–vis absorption spectra of ABTS solution incubated with different concentrations of MZG nanoparticles for 5 min (a sample picture is shown in the inset, equivalent concentration of Myr: 0.25, 0.5, 1, and 2 µg·mL^−1^). (b) The scavenging rate of MZG nanoparticles as fitted by the maximum UV–vis absorption at 734 nm. (c) Stably scavenging radical activity evaluation of MZG nanoparticles and Myr/GSH complex (equivalent concentration of Myr: 4 μg·mL^−1^, ** indicates *p* < 0.01). (d) Evaluation of the sustainable radical scavenging activity of MZG nanoparticles (equivalent concentration of Myr: 4 μg·mL^−1^). (e) UV–vis absorption spectra of MZG nanoparticles treated with different concentrations of H_2_O_2_ (equivalent concentration of Myr: 20 μg·mL^−1^). (f) Size of MZG nanoparticles treated with different concentrations of H_2_O_2_ (equivalent concentration of Myr: 0.5 mg·mL^−1^).

In this work, the ABTS assay was employed to evaluate the radical scavenging activity [39]. The UV–vis absorption intensity of ABTS^+^ solution (0.7 mM) was 0.7 ± 0.02 in phosphate-buﬀered saline (PBS). The diluted ABTS^+^ solution was incubated with GSH, Myr, and MZG for accessing the radical scavenging activity. The diluted ABTS^+^ solution was incubated with different concentrations of Myr (0.5, 1, 2, 3, and 4 µg·mL^−1^) for 5 min. The color of the diluted ABTS^+^ solution gradually disappeared with increasing Myr concentration (Supporting Information File 1, Figure S2a). The scavenging rate of pure Myr at 4 µg·mL^−1^ was up to 94.0% (Supporting Information File 1, Figure S2b). In the same way, the scavenging rate of GSH was evaluated. The ABTS^+^ solution was treated with different concentrations of GSH (1, 2, 4, 6, and 8 µg·mL^−1^), and the color gradually disappeared (Supporting Information File 1, Figure S2c). Figure S2d (Supporting Information File 1) shows that the scavenging rate of GSH at 8 µg·mL^−1^ was up to 93.3%. Figure 3a shows that the UV–vis absorbance of diluted ABTS^+^ solution incubated with different concentrations of MZG nanoparticles (equivalent concentration of Myr: 0.25, 0.5, 1, 1.5, and 2 µg·mL^−1^, equivalent concentration of GSH: 1, 2, 3, and 4 µg·mL^−1^) gradually decreased, suggesting that MZG nanoparticles scavenged radicals. When the concentration of MZG nanoparticles was equivalent to 2 µg·mL^−1^ of Myr and 4 µg·mL^−1^ of GSH, the scavenging rate of MZG was up to 93.5% (Figure 3b). This demonstrates that the scavenging effect of co-assembled MZG nanoparticles is identical to that of the Myr/GSH complex, indicating that co-assembly did not affect the ROS scavenging activity of Myr and GSH. However, as MZG nanoparticles could not only scavenge ROS, but also overcome the poor water solubility of Myr, the co-assembly was effective to enhance the bioavailability of both Myr and GSH, especially regarding a sustainable antioxidant efficacy. The stable radical scavenging activity of MZG was further assessed. As shown in Figure 3c, the radical scavenging activity of the Myr/GSH complex was lower than that of MZG nanoparticles at the same concentration over 5 days, which demonstrated that MZG nanoparticles were more stable to scavenge radicals than the Myr/GSH complex. Next, we explored whether the as-prepared MZG nanoparticles possessed the sustainability of radical scavenging activity due to the co-assembly that might have enhanced the stability of Myr and GSH. As shown in Figure 3d, ABTS^+^ solution was persistently added to 1 mL of MZG nanoparticle suspension or water for 5 days. The result revealed that MZG nanoparticles exhibited prolonged activity for scavenging ABTS^+^ compared with water. It demonstrated that the as-prepared MZG nanoparticles exhibited stable and sustainable radical scavenging activity.

ROS-responsive disruption of MZG nanoparticles during ROS scavenging was further explored. While MZG nanoparticles were incubated with diﬀerent concentrations of H_2_O_2_ (0.01, 0.1, 1, 10, and 100 mM), UV–vis absorption spectra and the size of the MZG nanoparticles were measured. The UV–vis absorbance of MZG nanoparticles from 300 to 400 nm (Figure 3e) decreased with the increase of H_2_O_2_ concentration. Also, the size of MZG nanoparticles was tested by DLS. The result showed that the size also decreased dramatically with the increase of H_2_O_2_ concentration. The UV–vis absorbance and size of MZG nanoparticles obviously changed at 1 mM of H_2_O_2_, indicating that there was a ROS-responsive disassembly of MZG nanoparticles (Figure 3f).

### Cell experiments

**Figure 4:**
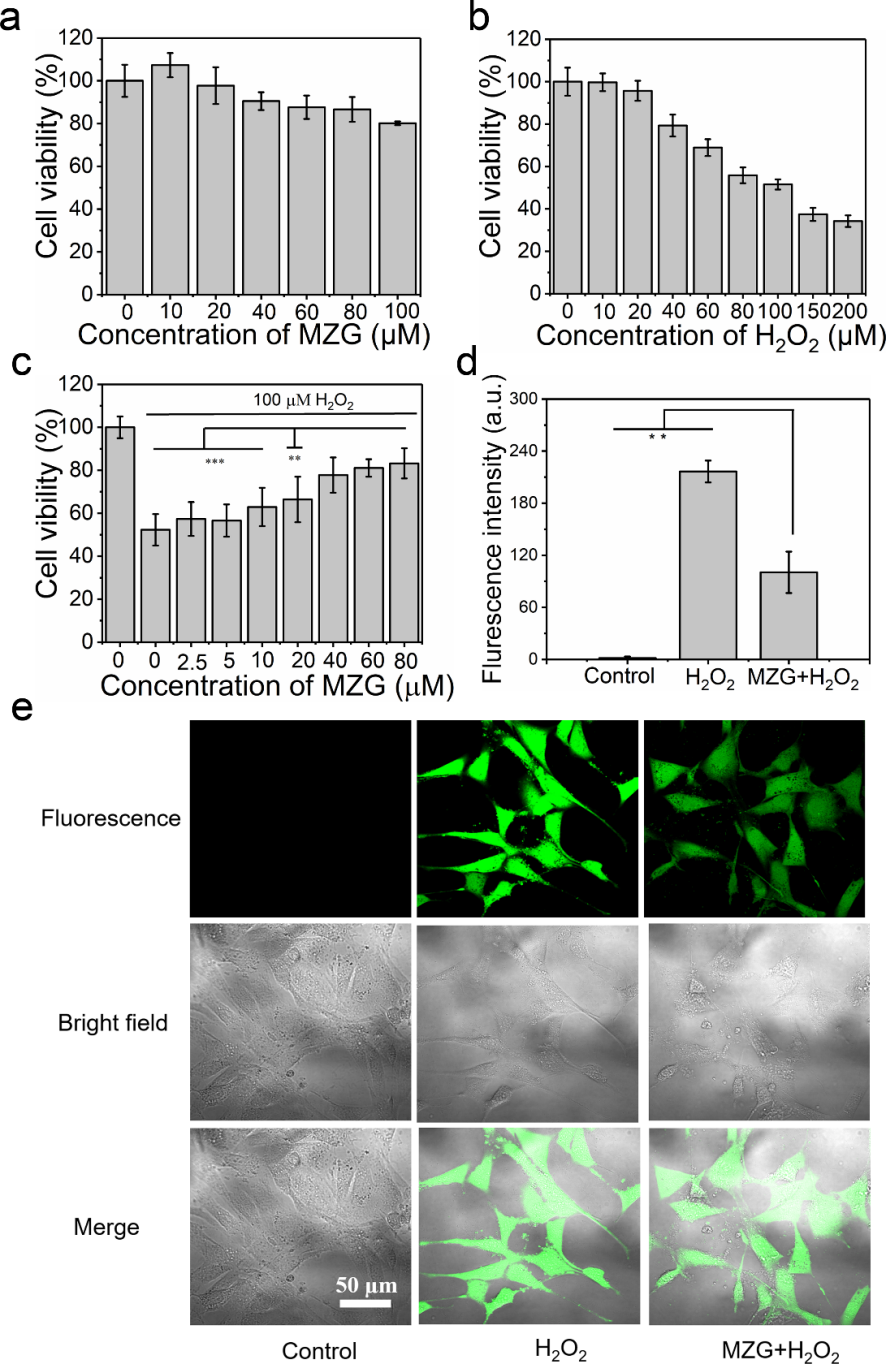
Antioxidant activity evaluation in cells. (a) Cytotoxicity evaluation of MZG nanoparticles by incubating 3T3 cells with different concentrations of MZG nanoparticles (equivalent concentration of Myr: 10, 20, 40, 80, and 100 µM). (b) H_2_O_2_-induced oxidative stress by incubating 3T3 cells with different concentrations of H_2_O_2_. (c) Antioxidant activity evaluation of MZG nanoparticles using 3T3 cells under H_2_O_2_-induced oxidative stress (** indicates *p* < 0.01, *** indicates *p* < 0.001). (d) Fluorescence intensity of ROS probed by DCFH-DA in cells after incubating with H_2_O_2_ and a combination of MZG nanoparticles and H_2_O_2_ (** indicates *p* < 0.01). (e) CLSM images of 3T3 cells probed by DCFH-DA after incubating with MZG nanoparticles and H_2_O_2_ (the first row shows fluorescence images, the second row shows bright-field images, and the third row shows the merged images).

The cytotoxicity of antioxidants is of importance for biomedical applications. Therefore, the cytotoxicity of MZG nanoparticles was assessed by incubating 3T3 cells and determining the cell viability via MTT assay [40]. 3T3 cells were treated with different concentrations of MZG nanoparticles (equivalent concentration of Myr: 10, 20, 40, 80, and 100 µM) for 24 h. The lowest cell viability was approximately 80% at the highest tested concentration of MZG nanoparticles (Figure 4a). The result indicates that MZG nanoparticles did not affect the growth of 3T3 cells.

The antioxidant activity of MZG nanoparticles was also examined using 3T3 cells and the MTT assay. H_2_O_2_ can produce ROS, and a high level of ROS can cause damage to cellular functions and components [41–42]. To explore the antioxidative effect of MZG nanoparticles the LD_50_ value of H_2_O_2_ against 3T3 cells was measured by the MTT assay. The cell viability of 3T3 cells decreased with increasing H_2_O_2_ concentration (Figure 4b). The LD_50_ concentration of H_2_O_2_ against 3T3 cells was 100 µΜ. Next, cell recovery due to the antioxidant activity of MZG nanoparticles was demonstrated by the MTT assay. After incubating with different concentrations of MZG nanoparticles (equivalent concentration of Myr: 2.5, 5, 10, 20, 40, and 80 µM) for 24 h, 3T3 cells were further incubated with 100 µΜ of H_2_O_2_ for 24 h. The cell viability gradually increased with increasing MZG concentration (Figure 4c).

This observation indicated that MZG could scavenge ROS to effectively protect cells from damage. DCFH-DA was used to probe ROS in cells, which showed no fluorescence signal without ROS, while it turned to highly fluorescent 2′7′-dichlorofluorescein after interacting with ROS in cells. As shown in the confocal laser scanning microscopy (CLSM) images, the fluorescence intensity of cells treated with H_2_O_2_ and MZG nanoparticles was weaker than that of cells only treated with H_2_O_2_ (Figure 4e). The fluorescence intensity of cells was further measured, indicating that the fluorescence intensity of cells treated with a combination of MZG nanoparticles and H_2_O_2_ was lower than that of cells treated with H_2_O_2_, suggesting that MZG nanoparticles were capable of scavenging ROS in cells (Figure 4d).

## Conclusion

We have prepared antioxidant MZG nanoparticles by co-assembly of the naturally occurring flavonoid Myr and GSH in the presence of Zn^2+^. The resulting MZG nanoparticles overcame the disadvantages of water-insoluble Myr and GSH that is quickly metabolized, improving their bioavailability. Importantly, the as-prepared MZG nanoparticles exhibited robust stability and sustainable ROS scavenging activity, protecting cells from ROS damage. The MZG nanoparticles provide an alternative opportunity for optimizing the antioxidation capability of conventional drugs and present great potential for further biomedical applications.

## Supporting Information

File 1 Additional experimental data.
