# Correction: Coordination-assembled myricetin nanoarchitectonics for sustainably scavenging free radicals

**DOI:** 10.3762/bjnano.13.48

**Published:** 2022-06-30

**Authors:** Xiaoyan Ma, Haoning Gong, Kenji Ogino, Xuehai Yan, Ruirui Xing

**Affiliations:** 1 Graduate School of Bio-Applications and Systems Engineering, Tokyo University of Agriculture and Technology, Tokyo, Japan; 2 State Key Laboratory of Biochemical Engineering, Chinese Academy of Sciences, Institute of Process Engineering, Beijing, P. R. China; 3 School of Chemical Engineering, University of Chinese Academy of Sciences, Beijing, P. R. China

**Keywords:** antioxidant, co-assembly, glutathione, myricetin, nanoarchitectonics

## Correction 1:

In the abbreviation of the radical cation "ABTS^+^", the dot indicating the radical molecule is missing. It should read "ABTS^•+^".

## Correction 2:

The sentence

“The change of DLS was recorded at different time points (0, 2, 4, 8, 24, 48, and 72 h), showing that the average size and size distribution did not change over time (Figure 1e,f).”

should read

“The change of DLS was recorded at different time points (0, 2, 4, 8, 24, 48, and 72 h), showing that the average size and size distribution did not change over time (Figure 2e,f)."

