# Supplementary material for: Coordination-assembled myricetin nanoarchitectonics for sustainably scavenging free radicals
Source: Beilstein J Nanotechnol. 2022 Mar 1;13:284–91. doi: 10.3762/bjnano.13.23 (PMC8895033; doi:10.3762/bjnano.13.23)
Supplement: File 1 — Additional experimental data. [file Beilstein_J_Nanotechnol-13-284-s001.pdf]

BEILSTEIN JOURNAL OF NANOTECHNOLOGY

Supporting Information

for

Coordination-assembled myricetin nanoarchitectonics for sustainably scavenging free radicals

Xiaoyan Ma, Haoning Gong, Kenji Ogino, Xuehai Yan and Ruirui Xing

Beilstein J. Nanotechnol. **2022,** *13,* 284–291. doi:10.3762/bjnano.13.23

Additional experimental data

License and Terms: This is a supporting information file under the terms of the Creative Commons Attribution License (https://creativecommons.org/licenses/by/4.0). Please note that the reuse, redistribution and reproduction in particular requires that the author(s) and source are credited and that individual graphics may be subject to special legal provisions.
The license is subject to the *Beilstein Journal of Nanotechnology* terms and conditions: (https://www.beilstein-journals.org/bjnano/terms)

Materials and Instruments

Materials: Myricetin (95%) was bought from Thermo Fisher Scientific. L-Glutathione (reduced) was obtained from Solarbio. Zinc(II) chloride ($ZnCl_2$) (98%) was purchased from Beijing Chemical Works. 2,2′-Azinobis-(3-ethylbenzthiazoline-6-sulfonate) was bought from Innochem (Beijing). 2′,7′-Dichlorodihydrofluorescein diacetate was bought from MedChemexpress. Dulbecco's Modified Eagle medium, fetal bovine serum (FBS), penicillin/ streptomycin, and trypsin-EDTA were bought from BioLegend.

Instruments: TEM images of nanoparticles were exhibited by a model JEM-1011 transmission scanning electron microscope (JEM-1011, JEOL, Japan). The size distribution and zeta potential of nanoparticles were measured by a Malvern DLS instrument (Zetasizer Nano ZS ZEN3600). UV/vis absorption spectra were tested by a Shimadzu UV-2600 spectrophotometer. FTIR spectra were obtained by a TENSOR 27 FTIR spectrometer (BRUKER). The cell viability was evaluated using MTT assay, in which the absorbance was measured by a microplate reader (Multiskan FC, Thermo Fisher Scientific). Confocal laser scanning microscopy (CLSM) images of cells were captured by CLSM (Olympus FV1000). Inductively coupled plasma-optical emission spectroscopy (ICP-OES, Prodigy, Leeman, USA) was used for evaluating concentration of Zn^{2+} in quantitative component analysis of MZG.

Table S1: Component analysis of MZG NPs.

Component	Myr	GSH	Zn^{2+}
Feeding concentration (mM)	3	6	1.57
Measured concentration (mM)	1.5	1.5	1.3

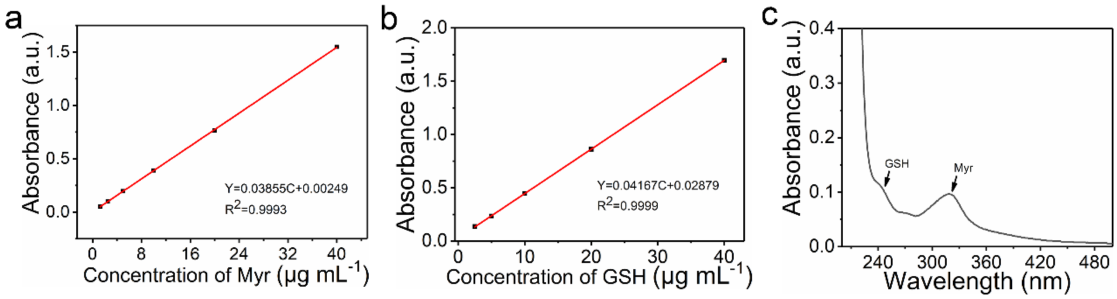

Figure S1: (a) The standard curve of Myr in 0.1 M NaOH. (b) The standard curve of GSH in 0.1 M NaOH. GSH dissolved in 0.1 M NaOH has an UV–vis absorption peak at ca. 240 nm. (c) The UV–vis absorption spectrum of diluted MZG nanoparticles (2000-fold) in 0.1 M NaOH.

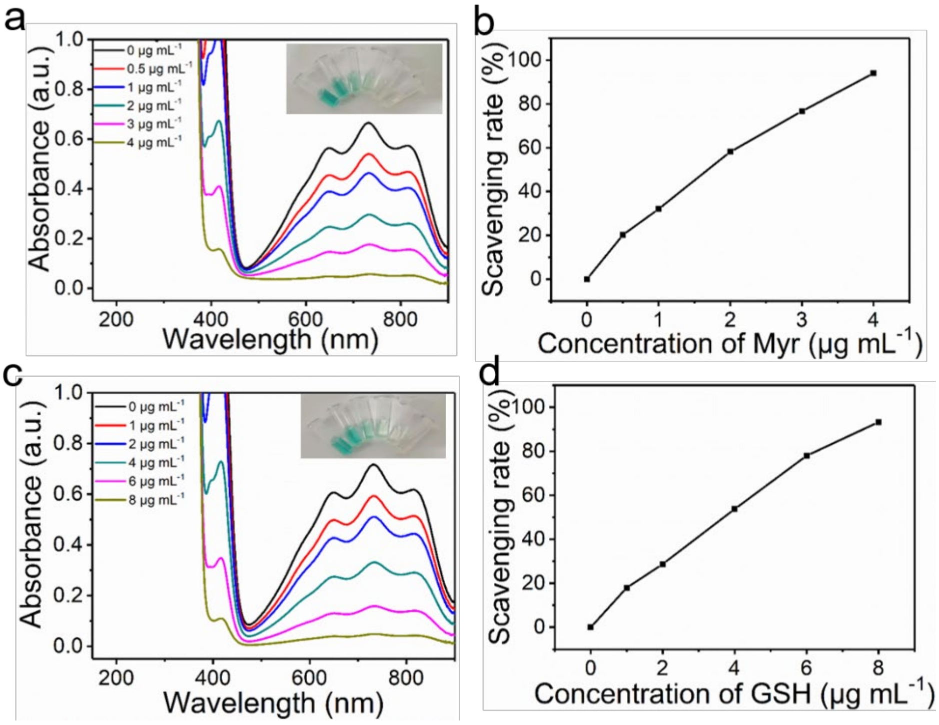

Figure S2: (a) UV–vis absorption spectra of ABTS solution incubated with different concentrations of Myr. A sample picture is shown in the inset. (b) Scavenging rate of Myr. (c) UV–vis absorption spectra of ABTS solution incubated with different concentrations of GSH. The inset shows a sample picture. (d) Scavenging rate of GSH.
